# Factors associated with in-hospital mortality in necrotising soft tissue infections. a multicentre retrospective cohort study

**DOI:** 10.1007/s00068-026-03242-0

**Published:** 2026-06-12

**Authors:** Mauro Podda, Marco Ceresoli, Francesco Virdis, Fausto Rosa, Tiziana Pilia, Valentina Murzi, Alessia Dessì, Silvia Tedesco, Caterina Cina, Pasquale Chiacchio, Carla Vitiello, Emilio Paolo Emma, Stefano Piero Bernardo Cioffi, Michele Altomare, Stefania Cimbanassi, Adolfo Pisanu

**Affiliations:** 1 Department of Surgical Science, Emergency Surgery Unit, Policlinico Universitario di Monserrato, University of Cagliari, Cagliari, Italy; 2 School of Medicine and Surgery, University of Milano-Bicocca, Milan, Italy; 3 General and Emergency Surgery, Fondazione IRCCS San Gerardo dei Tintori, Monza, Italy; 4 Acute Care and Trauma Surgery Unit, ASST GOM Niguarda, Milan, Italy; 5 Department of Emergency and Trauma Surgery, Università Cattolica del Sacro Cuore, Roma, Italy; 6 Department of Surgical Science, University of Cagliari, Cagliari, Italy

**Keywords:** Necrotizing soft tissue infections, Necrotizing fasciitis, Fournier’s gangrene, Mortality, Risk prediction

## Abstract

**Purpose:**

Necrotising soft tissue infections (NSTIs) are rare but life-threatening conditions associated with high mortality rates. This multicentre study aimed to identify admission variables associated with in-hospital mortality.

**Methods:**

This retrospective study included adult patients with surgically confirmed NSTIs treated at four high-volume academic referral centres in Italy between 2010 and 2024. Demographic, clinical, physiological, and laboratory variables available at hospital admission were analysed. Categorical variables were compared between survivors and non-survivors using the chi-square test or Fisher’s exact test, while quantitative variables were compared using the Student’s t test or Mann–Whitney U test. Multivariable logistic regression analyses were performed to identify independent predictors of in-hospital mortality. Results were reported as ORs with 95%CI. A prognostic nomogram was developed from the final multivariable model, including variables independently associated with mortality.

**Results:**

A total of 379 patients were included. In-hospital mortality was 16.7%. In subgroup comparisons, mortality was 17.0% in necrotising fasciitis and 22.4% in Fournier’s gangrene (*P* = 0.275). In the overall NSTI population, age (aOR 1.061, 95%CI 1.037–1.087) and serum creatinine at admission (aOR 1.301, 95%CI 1.040–1.629) were independently associated with mortality. In subgroup analyses, age (aOR 1.079, 95%CI 1.051–1.114) and chronic kidney disease (aOR 2.885, 95%CI 1.141–7.283) remained associated with mortality in limb necrotizing fasciitis, while only age (aOR 1.074, 95%CI 1.030–1.124) remained independently associated with mortality in Fournier’s gangrene. However, subgroup analyses in necrotising fasciitis of the limbs and Fournier’s gangrene were limited by low event rates and should be considered exploratory. The nomogram based on age and serum creatinine predicted in-hospital mortality (AUC 0.775, 95% CI 0.711–0.831), with good agreement between predicted and observed outcomes across risk levels.

**Media summary:**

The FATAL-NSTI study identified age and serum creatinine at admission as key predictors of in-hospital mortality in necrotising soft tissue infections. A nomogram based on age and serum creatinine may support early risk stratification. #NecrotisingSoftTissuesInfections; #FournierGangrene; #NecrotisingFasciitis

**Conclusions:**

NSTIs remain associated with substantial mortality. Age was the most consistent predictor of in-hospital mortality.

**Supplementary Information:**

The online version contains supplementary material available at 10.1007/s00068-026-03242-0.

## Background

Necrotizing soft tissue infections (NSTIs) are rare but life-threatening conditions characterized by rapid tissue destruction, severe systemic inflammation, and a high risk of sepsis and multiorgan failure [1, 2]. Although they most commonly affect the extremities and trunk, any anatomical site may be involved. Fournier’s gangrene represents a particularly aggressive form, involving the perineal and genital regions, predominantly in men [3–6]. NSTIs can be polymicrobial, typically involving mixed aerobic and anaerobic flora, or monomicrobial, most often caused by toxin-producing *Streptococcus pyogenes* [7, 8].

Despite advances in critical care and surgical management, mortality remains high, ranging from 10% to 45%, and exceeding 50% in patients with organ failure [6, 9, 10]. Rapid disease progression, sometimes advancing by several centimetres per hour, makes early recognition crucial [11, 12]. However, diagnosis is frequently delayed, as early clinical features are often nonspecific and overlap with those of more common soft tissue infections, leading to high rates of initial misdiagnosis [13].

Prompt and aggressive surgical debridement is the cornerstone of treatment and is consistently associated with improved survival [14]. Several patient- and disease-related factors, including advanced age, diabetes, cardiovascular and renal disease, malignancy, and immunosuppression, have been linked to worse outcomes [15–21]. Although laboratory-based tools such as the LRINEC score have been proposed to support early diagnosis and risk stratification, their clinical value remains controversial [22–24].

Among survivors, morbidity is substantial. Limb amputation is required in up to 15% of cases, and many patients experience long-term functional impairment and reduced quality of life, with significant implications for rehabilitation and healthcare resources [25–29]. Current evidence is limited by small, heterogeneous cohorts and a lack of focus on variables available at hospital admission that could guide early clinical decision-making [30].

The present multicentre retrospective study aimed to identify early predictors of in-hospital mortality in a large cohort of patients with surgically confirmed NSTIs treated at four high-volume academic centres in Italy.

## Methods

### Study design

This multicentre retrospective observational study was conducted at four high-volume tertiary referral academic hospitals in Italy, all serving as regional reference centres for the management of NSTIs. Participating institutions included the Emergency Surgery Unit of Cagliari University Hospital (Cagliari), the Trauma Team & Emergency Surgery Unit of Niguarda Hospital (Milan), the General & Emergency Surgery Unit of San Gerardo dei Tintori Hospital (Monza), and the Emergency Surgery & Trauma Unit of Policlinico Universitario A. Gemelli IRCCS (Rome). Consecutive patients treated between January 2010 and December 2024 were included. Follow-up was performed until hospital discharge or death. Data were collected retrospectively between May and June 2025 through review of institutional operative registries and electronic medical records. Data extraction was completed in July 2025, and statistical analyses were performed in October 2025.

The research was conducted in accordance with the Declaration of Helsinki and current regulations on good clinical practice. The study was designed and reported in accordance with the STROBE (Strengthening the Reporting of Observational Studies in Epidemiology) guidelines [31]. Approval for study conduct was obtained from the institutional review boards of the participating centres. Due to the retrospective design, the requirement for informed consent was waived according to Italian regulations. The study protocol was registered on ClinicalTrials.gov (NCT07359651. Registration date: 2026-01-22).

### Eligibility criteria

Eligible patients were adults aged ≥ 18 years with a diagnosis of NSTI confirmed at surgical exploration. Exclusion criteria were inability to complete in-hospital follow-up or incomplete clinical records precluding assessment of the primary outcome. For analytical purposes, subgroup analyses were performed for necrotizing fasciitis of the limbs and Fournier’s gangrene.

### Outcomes

The primary outcome measure was in-hospital mortality, defined as death occurring during the index hospitalization.

### Variables

Collected variables included demographic data, comorbidities, clinical presentation at admission, vital signs, laboratory parameters obtained at admission, microbiological findings, and treatment-related variables. Data were extracted from electronic medical records, operative reports, laboratory databases, and discharge notes. All measurements reflected routine clinical practice. Diagnostic and therapeutic pathways for NSTIs were homogeneous and similar across participating centres, according to existing guidelines [32, 33].

Selection bias was minimized by including consecutive eligible patients over the study period, through the search of standardized institutional medical records, and restricting inclusion to surgically confirmed NSTIs. Potential confounding was addressed through multivariable regression analysis adjusting for clinically relevant variables.

### Statistical analysis

Normality of quantitative variables was assessed using the Shapiro–Wilk test. Variables were summarised as median with interquartile range (Q1-Q3, IQR), according to data distribution. Clinically relevant thresholds were explored using receiver operating characteristic (ROC) analysis and the Youden index. These thresholds were derived post hoc for clinical interpretability and were intended for descriptive purposes only.

Inferential analyses compared non-survivors and survivors. Comparisons were performed in the overall NSTIs cohort and separately within each predefined subgroup. Categorical variables were compared using the Chi-square test or Fisher’s exact test, as appropriate. Continuous variables were compared using the Mann–Whitney U test, according to data distribution.

Variables statistically associated with the outcome at univariable analysis (*P* < 0.05) were entered into multivariable logistic regression models to identify independent predictors of in-hospital mortality. Results were reported as odds ratios (ORs) with 95% confidence intervals (95%CI). Model discrimination was assessed using the area under the receiver operating characteristic curve (AUC). Internal validation was performed using bootstrap resampling, and model calibration was evaluated using calibration plots, calibration intercept, and slope.

A prognostic nomogram for in-hospital mortality was subsequently developed based on the final multivariable model. Missing data were handled using complete-case analysis and multiple imputation, with imputed datasets used for multivariable analyses. The association between year of admission and in-hospital mortality was assessed using univariable logistic regression, modelling year as a continuous variable. All tests were two-sided, and *P* < 0.05 was considered statistically significant. Statistical analyses were performed using jamovi (version 2.7.9.0; www.jamovi.org).

## Results

**Fig. 1:**
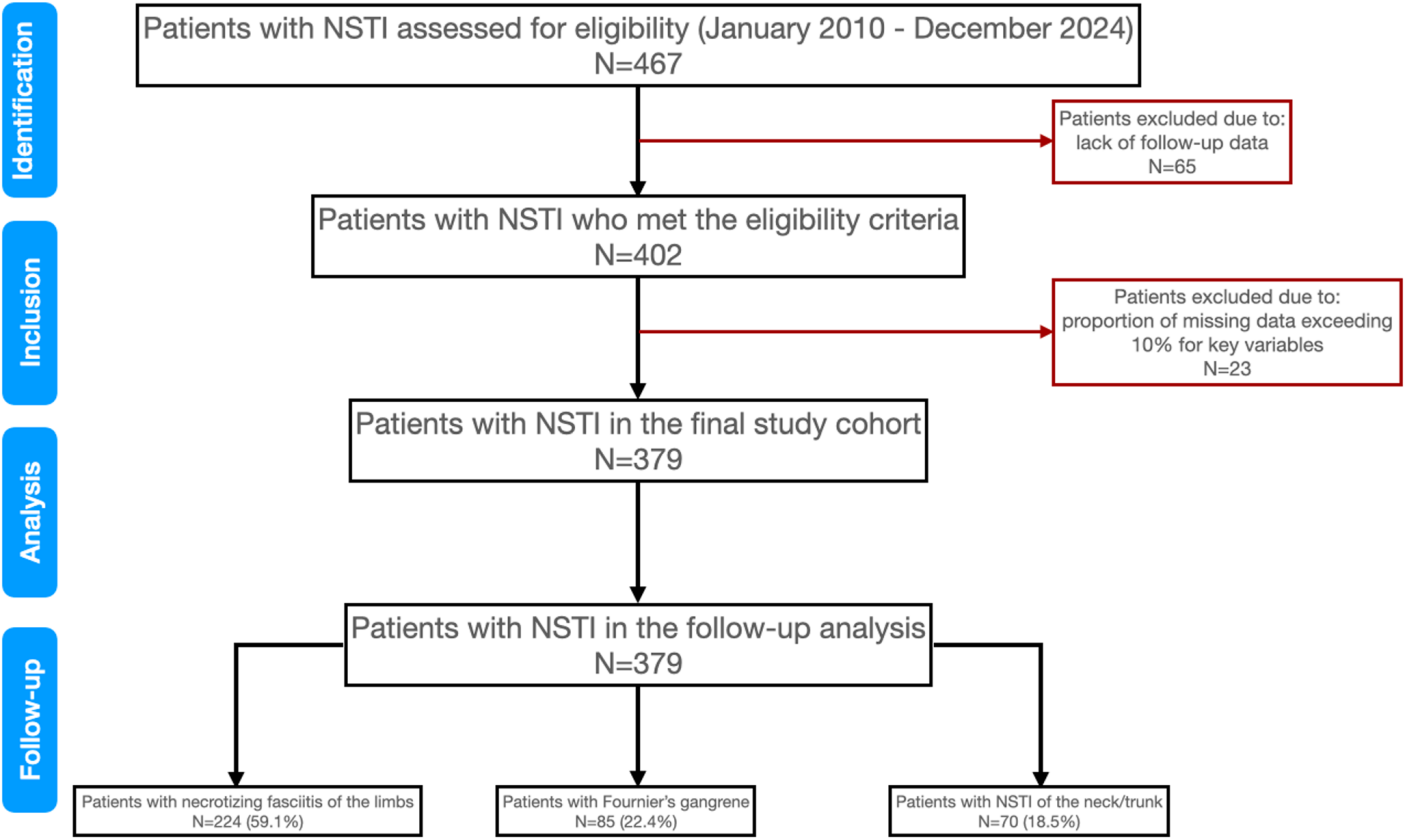
Flow diagram of patient selection

A total of 467 patients were screened from institutional records, and 402 initially met the inclusion criteria. Twenty-three patients (10%) were excluded due to missing data exceeding 10% for key variables. A final cohort of 379 patients was included in the analysis (Fig. 1). Necrotizing fasciitis of the limbs was the most common presentation (59.1%), followed by Fournier’s gangrene (22.4%) and NSTIs of the neck and trunk (18.5%). Median age was 60 years (40.0–70.0, IQR 23), and 65.2% were male. Hypertension (44.9%) and diabetes mellitus (39.6%) were the most frequent comorbidities. At admission, patients showed marked systemic inflammation, with median WBC 14.6 × 10⁹/L (9.4–20.3, IQR 10.8), CRP 194.5 mg/L (102.8-290.3, IQR 189.8), procalcitonin 2.7 ng/mL (0.8–11.7, IQR 11.2), and a median LRINEC score of 6.0 (3.0–8.0, IQR 5.0). Polymicrobial infections were common. The most frequently isolated pathogens included *Escherichia coli* (20.6%), *Streptococcus anginosus* group (13.7%), *Enterococcus* spp. (11.9%), and *Staphylococcus aureus* (6.6%). Multidrug-resistant organisms were identified in 6.9% of cases. Broad-spectrum antimicrobial therapy was routinely administered, most commonly including piperacillin–tazobactam (56.2%), meropenem (28.5%), and daptomycin (29.0%).

**Fig. 2:**
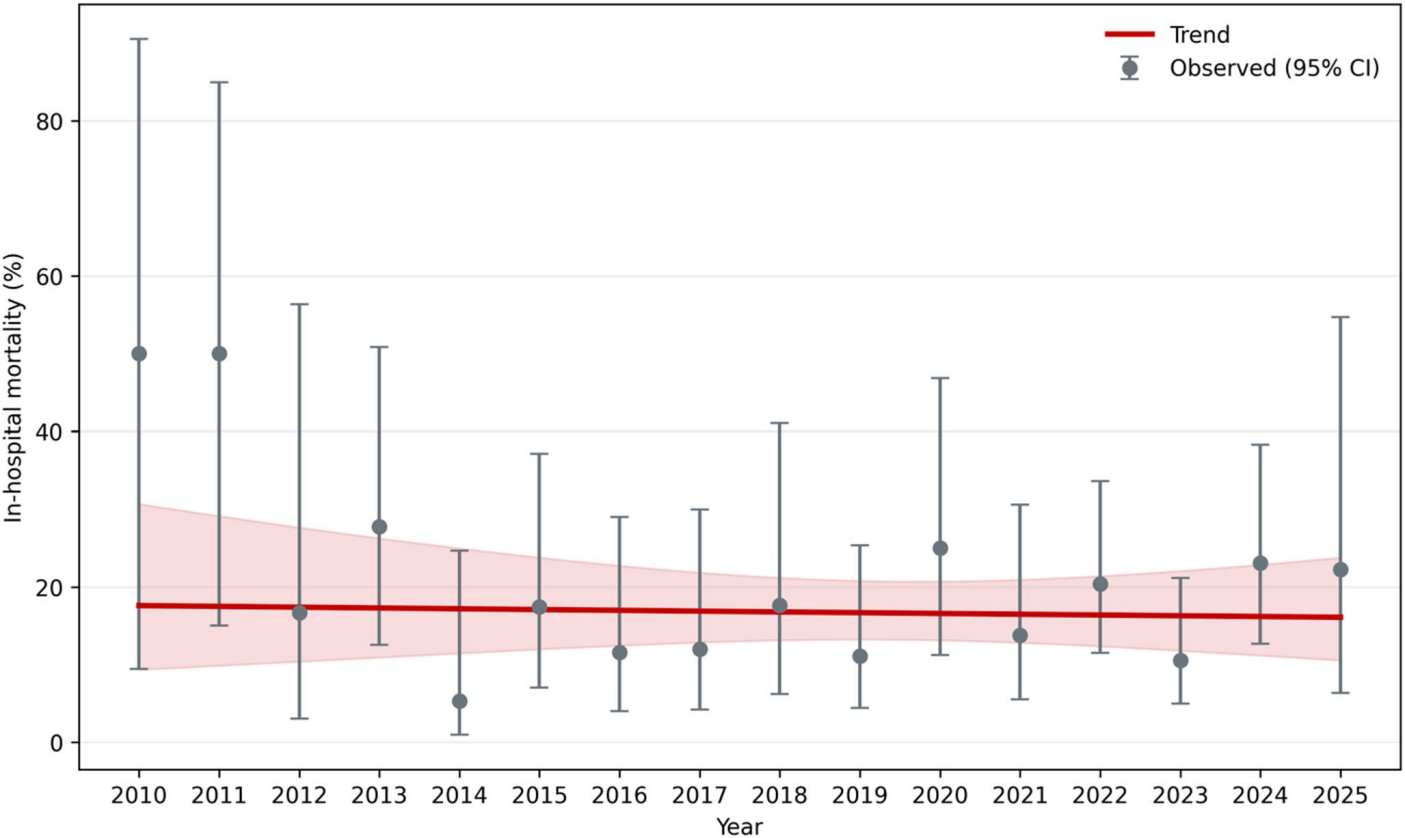
Temporal trend in in-hospital mortality across study years

**Fig. 3:**
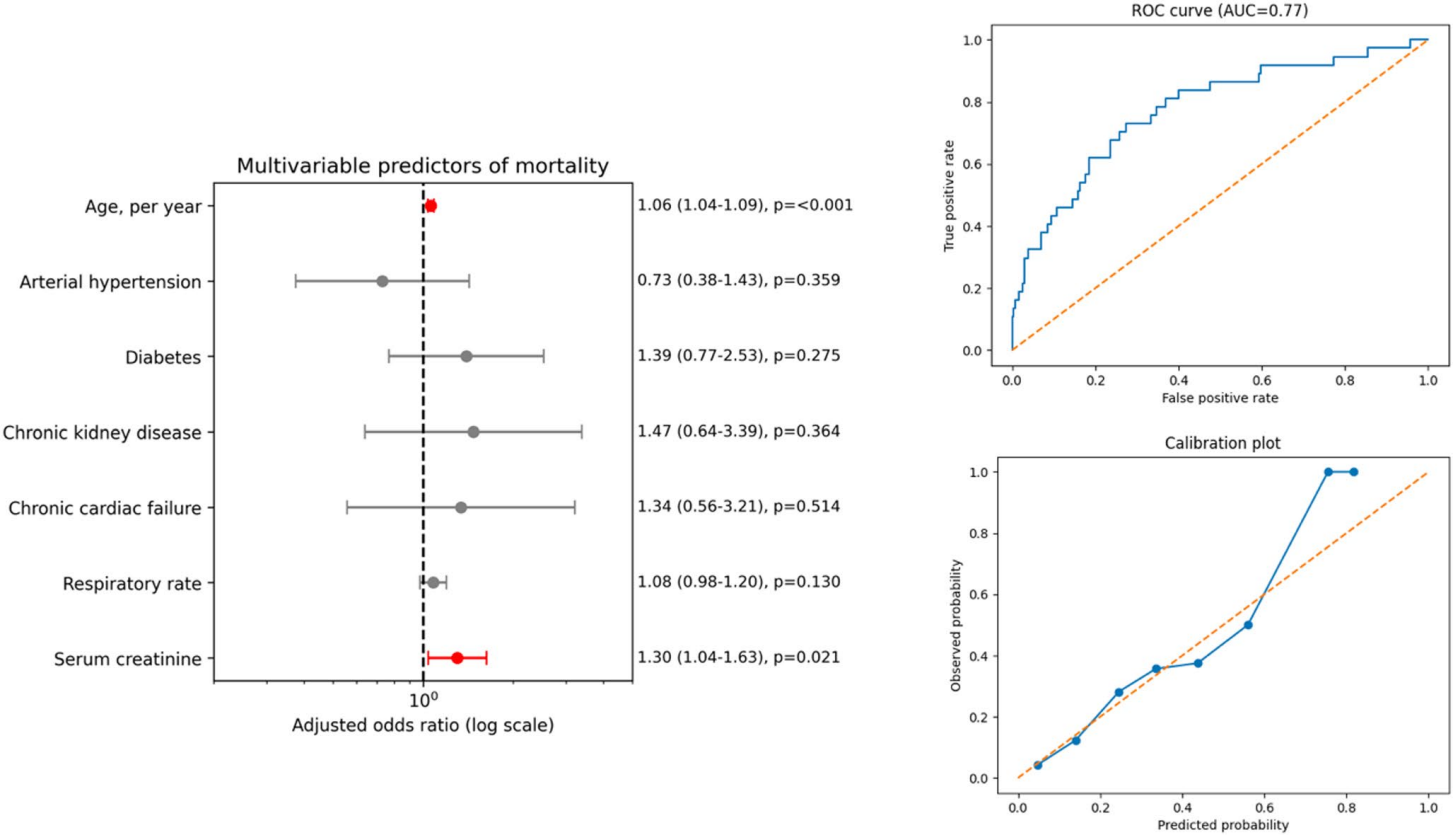
Multivariable analysis and calibration of the in-hospital mortality prediction model in the overall NSTI population

**Fig. 4:**
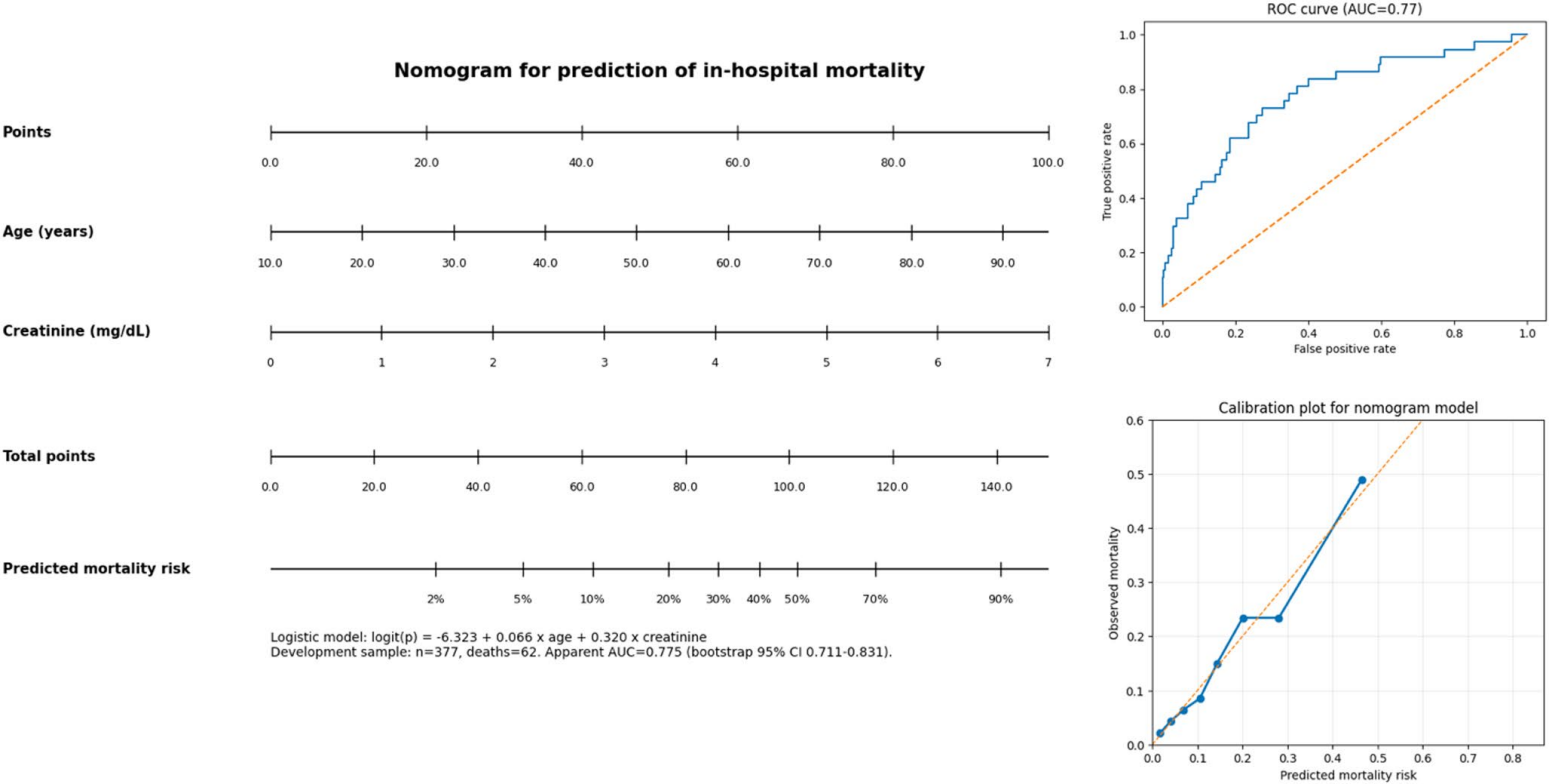
Nomogram for prediction of in-hospital mortality and model calibration in the overall NSTI population

The median duration of symptoms before hospital admission was 6.0 days (3.0–10.0, IQR 7.0). During hospitalization, patients underwent a median of three surgical revisions (2.0–4.0, IQR 2.0); 12.9% of patients were submitted to faecal diversion, and 45.6% were treated with negative pressure wound therapy (NPWT). The median length of hospital stay was 22.0 (12.0–44.0, IQR 32.0) days.

Baseline characteristics of the study population are reported in Table 1. Characteristics and outcomes among the two subgroups, necrotizing fasciitis of the limbs and Fournier’s gangrene, were reported in Supplementary Tables 1–2.

### Outcomes’ analysis

In the overall NSTIs cohort, in-hospital mortality occurred in 16.7% of patients. Patients underwent a median of three surgical debridements (2.0–4.0, IQR 2.0), and median length of hospital stay was 22 days (12.0–44.0, IQR 32.0). In subgroup analyses, mortality was 17.0% in necrotizing fasciitis of the limbs and 22.4% in Fournier’s gangrene (*P* = 0.275). The median number of surgical debridements was three in both groups (1.0–4.0, IQR 3.0 vs. 2.0–5.0, IQR 3.0; *P* = 0.310), while median length of hospital stay was 22 (12.0–45.0, IQR 33.0) and 27 days (15.0–46.0, IQR 31.0), respectively (*P* = 0.180) (Table 2). Despite year-to-year variability, no significant temporal trend in in-hospital mortality was observed over the study period (OR per year 0.99, 95% CI 0.92–1.07; *P* = 0.846) (Fig. 2).

### Predictors of in-hospital mortality

Univariable analysis identified several variables associated with in-hospital mortality, including age, arterial hypertension, diabetes, chronic kidney disease, chronic cardiac failure, respiratory rate, procalcitonin, and serum creatinine (Table 3).

In multivariable analysis, only age (adjusted OR [aOR] 1.061 per year, 95%CI 1.037–1.087; *P* < 0.001) and serum creatinine at admission (aOR 1.301 per mg/dL, 95%CI 1.040–1.629; *P* = 0.021) remained independently associated with mortality. The model showed good discrimination (AUC 0.775, 95% CI 0.711–0.831) (Fig. 3).

Clinically relevant thresholds for age and serum creatinine were explored post hoc using the Youden index (62 years and 1.6 mg/dL, respectively) and are reported for descriptive purposes only.

In subgroup analyses, age remained independently associated with in-hospital mortality in both necrotizing fasciitis of the limbs (aOR 1.079 per year, 95%CI 1.051–1.114; *P* < 0.001) and Fournier’s gangrene (aOR 1.074 per year, 95%CI 1.030–1.124; *P* = 0.002).

In patients with necrotizing fasciitis of the limbs, chronic kidney disease was also independently associated with mortality (aOR 2.885, 95%CI 1.141–7.283; *P* = 0.025). In Fournier’s gangrene, no additional variables were independently associated with mortality after multivariable analysis (Supplementary Table 3). Subgroup analyses in necrotizing fasciitis of the limbs and Fournier’s gangrene were limited by small event numbers and should be considered exploratory and interpreted with caution.

Clinically relevant thresholds for age were explored post hoc using the Youden index (≥ 61 and ≥ 74 years, respectively) and are reported for descriptive purposes only.

### Nomogram

A prognostic nomogram demonstrated good discrimination, with an AUC of 0.775 (95% CI 0.711–0.831) and a bootstrap-corrected AUC of 0.771. Calibration analysis showed minimal global miscalibration, with a calibration intercept close to 0 and a calibration slope close to 1. Bootstrap-based calibration curves confirmed stable agreement between predicted and observed mortality across the range of predicted risks (Fig. 4).

## Discussion

The risk of in-hospital death associated with NSTIs has decreased over time but remains substantial [1]. In our study, in-hospital mortality in the general cohort of patients with NSTIs was 16.7%, consistent with contemporary reports from specialised centres [9, 14, 15, 22, 34, 35]. Increasing age and higher serum creatinine levels at admission were the only variables independently associated with mortality in the overall NSTI population. Although the association between age and mortality has been previously described in studies with limited sample size, our findings from a large multicentre cohort further support the consistency of age as an independent prognostic factor in NSTIs [20, 36]. Older patients often present with a higher burden of comorbidities, reduced physiological reserve, and impaired immune responses, which may limit their capacity to withstand the systemic inflammatory and metabolic stress associated with these infections [29]. Similarly, elevated serum creatinine reflects either pre-existing chronic kidney disease or early acute kidney injury, both of which are strongly associated with sepsis-related complications and mortality [18]. In our study, the identification of clinically meaningful cut-off values for age and creatinine using Youden’s index further supports their potential role in bedside risk stratification, although these thresholds should be interpreted cautiously and validated externally.

Subgroup analyses yielded additional, albeit more limited, insights. In patients with necrotizing fasciitis of the limbs, age and chronic kidney disease remained the sole independent predictors of mortality. In Fournier’s gangrene, only age was independently associated with death. Although subgroup analyses in necrotizing fasciitis of the limbs and Fournier’s gangrene should be considered exploratory due to limited event numbers and wide confidence intervals, the observed association with age is consistent with previous evidence indicating a poorer prognosis in older patients with severe infections [37–39].

An important finding of this study is the lack of prognostic value of the LRINEC score. Although LRINEC has been widely proposed as both a diagnostic and prognostic tool for NSTIs, its performance remains controversial [2]. In the present cohort, LRINEC was not independently associated with mortality, and demonstrated poor discriminatory ability for the analysed outcome. This result is consistent with growing evidence questioning the utility of LRINEC for prognostic stratification, particularly in high-acuity referral populations. They suggest that reliance on laboratory-based composite scores alone may be insufficient to capture the complexity and heterogeneity of NSTI severity.

Building on the multivariable mortality analysis, a prognostic nomogram was developed using variables selected on the basis of statistical significance. The nomogram showed good discrimination and stable calibration after internal bootstrap validation, with close agreement between predicted and observed mortality across the range of predicted risks. By integrating simple admission variables such as age and serum creatinine into a graphical tool, the nomogram may support early bedside estimation of mortality risk, facilitate prioritisation of intensive care resources, and improve communication with patients and families. It may also assist in identifying patients in whom aggressive treatment strategies are most likely to be beneficial, while helping to avoid potentially futile interventions. Nevertheless, the nomogram should be regarded as an adjunct to, rather than a replacement for, clinical judgement, and requires external validation before routine implementation.

While this study focused on in-hospital mortality, long-term functional outcomes represent a critical but underexplored dimension of NSTIs [40–42]. Future prospective studies incorporating standardized functional measures and patient-reported outcomes are needed to better define recovery trajectories and identify modifiable determinants of disability.

Several strengths of this study warrant consideration. The multicentre design and inclusion of high-volume tertiary referral centres enhance the internal validity of the findings within specialised healthcare settings. All cases were surgically confirmed, ensuring strict diagnostic accuracy. Uniform inclusion criteria reduced heterogeneity, and the focus on admission variables increased clinical applicability. Excluding registry-based analyses [15], this study represents one of the largest single cohorts reported in the literature. Moreover, all patients underwent surgical intervention within 12 h of hospital admission, in accordance with international recommendations, highlighting the importance of early source control once diagnosis is established [11].

Several limitations must be acknowledged. The retrospective design is inherently susceptible to residual confounding and information bias. Although consecutive patients were included and multivariable adjustment was performed, unmeasured variables may have influenced outcomes.

Multivariable analyses in subgroups were limited by low event rates, particularly in Fournier’s gangrene. This may have resulted in overfitting and limits the reliability and generalizability of subgroup-specific findings.

Data on lactate and base deficit were not available and were not included in the analysis. This may have limited the assessment of early metabolic derangement and risk stratification.

This study confirms that NSTIs continue to be associated with substantial mortality, even in specialised centres. Early admission variables, particularly age and serum creatinine, independently predict in-hospital mortality. Future prospective studies and external validation are required to refine our prognostic nomogram and improve outcome prediction in this complex and life-threatening condition.

**Table 1:** Baseline characteristics of the study population

Diagnosis	*N*.	Percentage	Missing data *N*. (%)
Total number of patients with NSTI	*N*. 379		
**Necrotising fasciitis of the limbs**	N. 224	% 59.1	-
Fournier’s gangrene	N. 85	% 22.4	-
NSTI of the neck/trunk	N. 70	% 18.5	-
**Type of NSTI**			
Type I	N. 214	% 56.3	-
Type II	N. 133	% 35.2	-
Type III	N. 14	% 3.7	-
Type IV	N. 18	% 4.8	-
**Microbial species**			
Escherichia coli	N. 78	% 20.6	-
Staphylococcus aureus	N. 25	% 6.6	-
Streptococcus anginosus group	N. 52	% 13.7	-
Enterococcus spp.	N. 45	% 11.9	-
Klebsiella pneumoniae	N. 22	% 5.8	-
Pseudomonas aeruginosa	N. 26	% 6.9	-
Multiresistant spp. (MRSA, ESBL, KPC)	N. 26	% 6.9	-
Candida albicans	N. 22	% 5.8	-
Candida glabrata	N. 8	% 2.1	-
**Antibiotics used (in combination)**			
Piperacillin-Tazobactam	N. 213	% 56.2	-
Linezolid	N. 70	% 18.5	-
Meropenem	N. 108	% 28.5	-
Clindamycin	N. 95	% 25.1	-
Daptomycin	N. 110	% 29.0	-
Metronidazole	N. 57	% 15.0	-
Vancomycin	N. 50	% 13.2	-
Amoxicillin/Clavulanic acid	N. 63	% 16.6	-
Ceftriaxone	N. 18	% 4.7	-
Teicoplanin	N. 13	% 3.4	-
Levofloxacin	N. 33	% 8.7	-
Colistin	N. 10	% 2.6	-
Ceftazidime/Avibactam	N. 5	% 1.3	-
Fluconazole	N. 22	% 5.8	-
Caspofungin	N. 10	% 2.6	-
Echinocandins	N. 1	% 0.3	-
**Age (years)**	Median 60.00	47.0–70.0 (IQR 23)	-
**Male sex**	N. 247	% 65.2	-
**Female sex**	N. 132	% 34.8	-
**Body Mass Index (BMI) Kg/m** ^**2**^	Median 25.00	22.5–29.0 (IQR 6.5)	5 (1.3%)
**Tobacco smoking (active)**	N. 107	% 28.2	-
**Alcohol consumption (active)**	N. 46	% 12.2	-
**Intravenous drug use (active)**	N. 38	% 10.1	-
**Arterial hypertensions**	N. 170	% 44.9	-
**Diabetes**	N. 150	% 39.6	-
**Ischaemic heart disease**	N. 81	% 21.4	-
**Peripheral neuropathy**	N. 33	% 8.7	-
**Active cancer disease**	N. 35	% 9.2	-
**Cirrhosis**	N. 28	% 7.4	-
**Chronic kidney disease**	N. 51	% 13.5	-
**Chronic liver disease**	N. 47	% 12.4	-
**Chronic respiratory failure**	N. 21	% 5.5	-
**Chronic cardiac failure**	N. 35	% 9.2	-
**Chronic obstructive pulmonary disease**	N. 12	% 3.2	3 (0.8%)
**Dermatological disease**	N. 25	% 6.6	-
**Haematological malignancies**	N. 31	% 8.2	-
**Chronic corticosteroid therapy**	N. 35	% 9.3	-
**Ongoing hypoglycaemic therapy**			
Oral hypoglycaemic therapy	59	% 15.6	-
Insulin	36	% 9.5	-
**Ongoing long-term NSAID therapy**	N. 41	% 10.8	-
**Recent surgery**	N. 46	% 12.1	-
**Duration of symptoms before hospital admission (days)**	Median 6.00	3.0–10.0 (IQR 7.0)	23 (6.1%)
**Body temperature at admission (°C)**	Median 37.30	36.5–38.2 (IQR 1.7)	2 (0.5%)
**Heart rate at admission (bpm)**	Median 95.00	80.0–110.0 (IQR 30.0)	1 (0.3%)
**Respiratory rate at admission (breaths/min)**	Median 15.50	13.0–18.0 (IQR 5.0)	34 (8.9%)
**Systolic blood pressure at admission (mmHg)**	Median 120.0	102.0-130.0 (IQR 27.7)	5 (1.3%)
**Diastolic blood pressure at admission (mmHg)**	Median 70.00	60.0–80.0 (IQR 20.0)	5 (1.3%)
**White Blood Cell (WBC) count at admission (x10^9/L)**	Median 14.59	9.4–20.3 (IQR 10.8)	4 (1.1%)
**Haemoglobin at admission (g/dL)**	Median 11.40	9.7–13.1 (IQR 3.4)	-
**Glycaemia at admission (mg/dL)**	Median 125.0	102.0-202.0 (IQR 100.0)	-
**C-reactive protein (CRP) at admission (mg/L)**	Median 194.5	102.8-290.3 (IQR 189.8)	25 (6.6%)
**Procalcitonin (PCT) at admission (ng/mL)**	Median 2.70	0.8–11.7 (IQR 11.2)	87 (22.9%)
**Serum sodium at admission (mEq/L)**	Median 137.0	133.0-140.0 (IQR 7.0)	-
**Serum creatinine at admission (mg/dL)**	Median 1.01	0.7–1.6 (IQR 0.9)	-
**LRINEC score at admission**	Median 6.00	3.0–8.0 (IQR 5.0)	14 (3.7%)

**Table 2:** Clinical outcomes of the general population and subgroup populations of patients with NSTIs

Clinical outcomes	General population	Necrotising fasciitis	Fournier’s gangrene	*P* Value
Death	N. 63 (16.7%)	N. 38 (17.0%)	N. 19 (22.4%)	*P* = 0.275
N. of surgical debridements	Median 3.00 (2.0–4.0, IQR 2.0)	Median 3.00 (1.0–4.0, IQR 3.0)	Median 3.00 (2.0–5.0, IQR 3.0)	*P* = 0.310
Length of hospital stay (days)	Median 22.00 (12.0–44.0, IQR 32.0)	Median 22.00 (12.0–45.0, IQR 33.0)	Median 27.00 (15.0–46.0, IQR 31.0)	*P* = 0.180

**Table 3:** Predictors of in-hospital mortality in the general populations of patients with NSTIs

	Odds Ratio/Mean Difference	95%CI	*P* value	Multivariable analysis
Age	1.069	1.051–1.093	< 0.001	aOR 1.061 per year, 95%CI 1.037–1.087, *P* < 0.001; Youden’s ≥62 years
Sex	1.092	0.622–1.917	0.759	
Body Mass Index (BMI) Kg/m^2^	1.014	0.145–1.173	0.865	
Tobacco smoking (active)	0.842	0.454–1.561	0.584	
Alcohol consumption (active)	1.093	0.483–2.474	0.831	
Intravenous drug use (active)	0.762	0.285–2.039	0.589	
Arterial hypertension	1.811	1.048–3.128	0.033	aOR 0.731, 95%CI 0.375–1.427, *P* = 0.359
Diabetes	2.021	1.171–3.489	0.011	aOR 1.394, 95%CI 0.768–2.533, *P* = 0.275
Ischaemic heart disease	1.606	0.871–2.963	0.129	
Peripheral neuropathy	0.671	0.227–1.980	0.470	
Active cancer disease	1.859	0.826–4.186	0.134	
Cirrhosis	1.407	0.546–3.624	0.480	
Chronic kidney disease	3.430	1.782–6.602	< 0.001	aOR 1.471, 95%CI 0.639–3.386, *P* = 0.364
Chronic liver disease	1.462	0.684–3.126	0.327	
Chronic respiratory failure	1.616	0.570–4.586	0.367	
Chronic cardiac failure	2.997	1.404–6.401	0.005	aOR 1.338, 95%CI 0.557–3.213, *P* = 0.514
Chronic obstructive pulmonary disease	1.759	0.462–6.692	0.408	
Dermatological disease	0.418	0.096–1.817	0.224	
Haematological malignancies	2.227	0.973–5.098	0.058	
Chronic corticosteroid therapy	1.617	0.696–3.757	0.264	
Oral hypoglycaemic therapy	1.721	0.177–2.182	0.114	
Insulin	0.602	0.205–1.766	0.355	
Ongoing long-term NSAID therapy	1.247	0.547–2.845	0.599	
Recent surgery	1.698	0.811–3.557	0.160	
Duration of symptoms before hospital admission (days)	0.996	0.982–1.011	0.621	
Body temperature at admission (°C)	0.904	0.708–1.150	0.419	
Heart rate at admission (bpm)	1.002	0.988–1.015	0.798	
Respiratory rate at admission (breaths/min)	1.123	1.033–1.220	0.006	aOR 1.082, 95%CI 0.977–1.197, *P* = 0.130
Systolic blood pressure at admission (mmHg)	0.990	0.978-1.000	0.085	
Diastolic blood pressure at admission (mmHg)	0.983	0.965-1.000	0.081	
White Blood Cell (WBC) count at admission (x10^9/L)	1.011	0.983–1.039	0.461	
Haemoglobin at admission (g/dL)	0.895	0.795–1.010	0.068	
Glycaemia at admission (mg/dL)	1.002	0.999–1.004	0.108	
C-reactive protein (CRP) at admission (mg/L)	1.001	0.999–1.003	0.236	
Procalcitonin (PCT) at admission (ng/mL)	1.009	1.000–1.019	0.049	aOR 1.000, 95%CI 0.990–1.020, *P* = 0.470
Serum sodium at admission (mEq/L)	0.974	0.928–1.020	0.277	
Serum creatinine at admission (mg/dL)	1.433	1.195–1.719	< 0.001	aOR 1.301 per mg/dL, 95%CI 1.040–1.629, *P* = 0.021, Youden’s ≥1.6 mg/dL
LRINEC score	1.083	0.989–1.186	0.085	

## Supplementary Information

Below is the link to the electronic supplementary material.

Supplementary Material 1

Supplementary Material 2

Supplementary Material 3

## Data Availability

Research data are available and will be provided by the corrisponding Author on reasonable request.

## References

[1] Hua C, Urbina T, Bosc R, Parks T, Sriskandan S, de Prost N, Chosidow O. Necrotising soft-tissue infections. Lancet Infect Dis. 2023;23:e81–94. 36252579 10.1016/S1473-3099(22)00583-7

[2] Fernando SM, Tran A, Cheng W, Rochwerg B, Kyeremanteng K, Seely AJE, Inaba K, Perry JJ. Necrotizing Soft Tissue Infection: Diagnostic Accuracy of Physical Examination, Imaging, and LRINEC Score: A Systematic Review and Meta-Analysis. Ann Surg. 2019;269:58–65. 29672405 10.1097/SLA.0000000000002774

[3] May AK, Talisa VB, Wilfret DA, Bulger E, Dankner W, Bernard A, Yende S. Estimating the Impact of Necrotizing Soft Tissue Infections in the United States: Incidence and Re-Admissions. Surg Infect (Larchmt). 2021;22:509–15. 32833599 10.1089/sur.2020.099

[4] McDermott J, Kao LS, Keeley JA, Grigorian A, Neville A, de Virgilio C. Necrotizing Soft Tissue Infections: A Review. JAMA Surg. 2024;159:1308–15. 39259555 10.1001/jamasurg.2024.3365

[5] Eke N. Fournier’s gangrene: a review of 1726 cases. Br J Surg. 2000;87:718–28. 10848848 10.1046/j.1365-2168.2000.01497.x

[6] El-Qushayri AE, Khalaf KM, Dahy A, Mahmoud AR, Benmelouka AY, Ghozy S, Mahmoud MU, Bin-Jumah M, Alkahtani S, Abdel-Daim MM. Fournier’s gangrene mortality: A 17-year systematic review and meta-analysis. Int J Infect Dis. 2020;92:218–25. 31962181 10.1016/j.ijid.2019.12.030

[7] Dhanasekara CS, Marschke B, Morris E, Kahathuduwa CN, Dissanaike S. Global patterns of necrotizing soft tissue infections: A systematic review and meta-analysis. Surgery. 2021;170:1718–26. 34362585 10.1016/j.surg.2021.06.036

[8] Hasham S, Matteucci P, Stanley PR, Hart NB. Necrotising fasciitis. BMJ. 2005;330:830–3. 15817551 10.1136/bmj.330.7495.830 PMC556077

[9] de Haan J, Neeter LMFH, Suijker J, van Zuijlen PPM, Pijpe A, Meij-de Vries A, NSTI Knowledge Collaborative Group. Characteristics, treatments, and outcomes of patients with necrotizing soft tissue infections: a Dutch multicenter cohort study. Eur J Trauma Emerg Surg. 2025;51:321. 10.1007/s00068-025-03000-8. 41148308 10.1007/s00068-025-03000-8 PMC12568877

[10] Sarani B, Strong M, Pascual J, Schwab CW. Necrotizing fasciitis: current concepts and review of the literature. J Am Coll Surg. 2009;208:279–88. 19228540 10.1016/j.jamcollsurg.2008.10.032

[11] Gelbard RB, Ferrada P, Yeh DD, Williams BH, Loor M, Yon J, Mentzer C, Khwaja K, Khan MA, Kohli A, Bulger EM, Robinson BRH. Optimal timing of initial debridement for necrotizing soft tissue infection: A Practice Management Guideline from the Eastern Association for the Surgery of Trauma. J Trauma Acute Care Surg. 2018;85:208–14. 29485428 10.1097/TA.0000000000001857

[12] Stevens DL, Bryant AE. Necrotizing Soft-Tissue Infections. N Engl J Med. 2017;377:2253–65. 29211672 10.1056/NEJMra1600673

[13] Bisgaard EK, Bulger EM. Current diagnosis and management of necrotizing soft tissue infections: What you need to know. J Trauma Acute Care Surg. 2024;97:678–86. 38689406 10.1097/TA.0000000000004351

[14] Vanguardia MKR, Lew C, Prabhakaran S, Kong JCH. Fournier’s gangrene: 15-year retrospective study at a tertiary hospital. BJS Open. 2024;8:zrae022. 10.1093/bjsopen/zrae022. 38626185 10.1093/bjsopen/zrae022 PMC11020226

[15] Hedetoft M, Madsen MB, Madsen LB, Hyldegaard O. Incidence, comorbidity and mortality in patients with necrotising soft-tissue infections, 2005–2018: a Danish nationwide register-based cohort study. BMJ Open. 2020;10:e041302. 10.1136/bmjopen-2020-041302. 33067303 10.1136/bmjopen-2020-041302 PMC7569942

[16] Lee CY, Kuo LT, Peng KT, Hsu WH, Huang TW, Chou YC. Prognostic factors and monomicrobial necrotizing fasciitis: gram-positive versus gram-negative pathogens. BMC Infect Dis. 2011;11:5. 10.1186/1471-2334-11-5. 21208438 10.1186/1471-2334-11-5 PMC3022716

[17] Eguia E, Vivirito V, Cobb AN, Janjua H, Cheung M, Kuo PC. Predictors of Death in Necrotizing Skin and Soft Tissue Infection. World J Surg. 2019;43:2734–9. 31312952 10.1007/s00268-019-05087-8 PMC6778025

[18] Bruun T, Rath E, Madsen MB, Oppegaard O, Nekludov M, Arnell P, Karlsson Y, Babbar A, Bergey F, Itzek A, Hyldegaard O, Norrby-Teglund A, Skrede S, INFECT Study Group. Risk Factors and Predictors of Mortality in Streptococcal Necrotizing Soft-tissue Infections: A Multicenter Prospective Study. Clin Infect Dis. 2021;72:293–300. 31923305 10.1093/cid/ciaa027 PMC7840107

[19] McCarty AR, Villarreal ME, Tamer R, Strassels SA, Schubauer KM, Paredes AZ, Santry H, Wisler JR. Analyzing Outcomes Among Older Adults With Necrotizing Soft-Tissue Infections in the United States. J Surg Res. 2021;257:107–17. 32818779 10.1016/j.jss.2020.06.031

[20] Horn DL, Shen J, Roberts E, Wang TN, Li KS, O’Keefe GE, Cuschieri J, Bulger EM, Robinson BRH. Predictors of mortality, limb loss, and discharge disposition at admission among patients with necrotizing skin and soft tissue infections. J Trauma Acute Care Surg. 2020;89:186–91. 32102045 10.1097/TA.0000000000002636 PMC7311238

[21] Nagira K, Ogoshi T, Akahori K, Enokida S, Enokida M, Ueda T, Homma M, Nagashima H. Factors associated with mortality in patients with extremity necrotizing soft-tissue infections: a single academic center experience. Langenbecks Arch Surg. 2023;408:189. 10.1007/s00423-023-02929-x. 37166568 10.1007/s00423-023-02929-x

[22] Kariksiz M, Ates O. Treatment and clinical outcomes in lower extremity necrotizing soft tissue infection. Eur J Trauma Emerg Surg. 2025;51:148. 10.1007/s00068-025-02835-5. 40126628 10.1007/s00068-025-02835-5

[23] Wong CH, Khin LW, Heng KS, Tan KC, Low CO. The LRINEC (Laboratory Risk Indicator for Necrotizing Fasciitis) score: a tool for distinguishing necrotizing fasciitis from other soft tissue infections. Crit Care Med. 2004;32:1535–41. 15241098 10.1097/01.ccm.0000129486.35458.7d

[24] Kim DY, Lavasile A, Kaji AH, Nahmias J, Grigorian A, Mukherjee K, Penaloza L, Posluszny J, Logan CD, Michelin E, Serena T, Sahr S, Bekdache K, Stoddard N, Choudhry A, Encalada RZ, Saltzman D, Padilla R, Truitt M, Grossman Verner H, Hunt D, Purvis V, Ross SW, Mallah MM, Dultz L, Kuhlenschmidt K, Mentzer CJ, Lonkar A, Chang G, Lemon B, de Virgilio C. Prospective derivation and validation of a necrotizing soft tissue infections score: An EASTmulticenter trial. J Trauma Acute Care Surg. 2024;97:910–7. 38720193 10.1097/TA.0000000000004374

[25] Cranendonk DR, van Vught LA, Wiewel MA, Cremer OL, Horn J, Bonten MJ, Schultz MJ, van der Poll T, Wiersinga WJ. Clinical Characteristics and Outcomes of Patients With Cellulitis Requiring Intensive Care. JAMA Dermatol. 2017;153:578–82. 28296993 10.1001/jamadermatol.2017.0159 PMC5817617

[26] Pham TN, Moore ML, Costa BA, Cuschieri J, Klein MB. Assessment of functional limitation after necrotizing soft tissue infection. J Burn Care Res. 2009;30:301–6. 19165118 10.1097/BCR.0b013e318198a241 PMC3042352

[27] de Prost N, Sbidian E, Chosidow O, Brun-Buisson C, Amathieu R, Henri Mondor Hospital Necrotizing Fasciitis Group. Management of necrotizing soft tissue infections in the intensive care unit: results of an international survey. Intensive Care Med. 2015;41:1506–8. 26109399 10.1007/s00134-015-3916-9

[28] McGillicuddy EA, Lischuk AW, Schuster KM, Kaplan LJ, Maung A, Lui FY, Bokhari SA, Davis KA. Development of a computed tomography-based scoring system for necrotizing soft-tissue infections. J Trauma. 2011;70:894–9. 21610394 10.1097/TA.0b013e3182134a76

[29] Brakenridge SC, Wilfret DA, Maislin G, Andrade KE, Walker V, May AK, Dankner WM, Bulger EM. Resolution of organ dysfunction as a predictor of long-term survival in necrotizing soft tissue infections: Analysis of the AB103 Clinical Composite Endpoint Study in Necrotizing Soft Tissue Infections trial and a retrospective claims database-linked chart study. J Trauma Acute Care Surg. 2021;91:384–92. 33797490 10.1097/TA.0000000000003183

[30] Altunok İ, Özdemir S. Enhanced prognostic model for mortality in patients with Fournier gangrene: Validation of the Fournier gangrene mortality index and integration of platelet count and PCO₂. Am J Emerg Med. 2025;100:170–4. 41380424 10.1016/j.ajem.2025.12.004

[31] von Elm E, Altman DG, Egger M, Pocock SJ, Gøtzsche PC, Vandenbroucke JP, STROBE Initiative. The Strengthening the Reporting of Observational Studies in Epidemiology (STROBE) statement: guidelines for reporting observational studies. J Clin Epidemiol. 2008;61:344–9. 18313558 10.1016/j.jclinepi.2007.11.008

[32] Sartelli M, Coccolini F, Kluger Y, Agastra E, Abu-Zidan FM, Abbas AES, Ansaloni L, Adesunkanmi AK, Augustin G, Bala M, Baraket O, Biffl WL, Ceresoli M, Cerutti E, Chiara O, Cicuttin E, Chiarugi M, Coimbra R, Corsi D, Cortese F, Cui Y, Damaskos D, de’Angelis N, Delibegovic S, Demetrashvili Z, De Simone B, de Jonge SW, Di Bella S, Di Saverio S, Duane TM, Fugazzola P, Galante JM, Ghnnam W, Gkiokas G, Gomes CA, Griffiths EA, Hardcastle TC, Hecker A, Herzog T, Karamarkovic A, Khokha V, Kim PK, Kim JI, Kirkpatrick AW, Kong V, Koshy RM, Inaba K, Isik A, Ivatury R, Labricciosa FM, Lee YY, Leppäniemi A, Litvin A, Luppi D, Maier RV, Marinis A, Marwah S, Mesina C, Moore EE, Moore FA, Negoi I, Olaoye I, Ordoñez CA, Ouadii M, Peitzman AB, Perrone G, Pintar T, Pipitone G, Podda M, Raşa K, Ribeiro J, Rodrigues G, Rubio-Perez I, Sall I, Sato N, Sawyer RG, Shelat VG, Sugrue M, Tarasconi A, Tolonen M, Viaggi B, Celotti A, Casella C, Pagani L, Dhingra S, Baiocchi GL, Catena F. WSES/GAIS/WSIS/SIS-E/AAST global clinical pathways for patients with skin and soft tissue infections. World J Emerg Surg. 2022;17:3. 10.1186/s13017-022-00406-2. 35033131 10.1186/s13017-022-00406-2 PMC8761341

[33] Sartelli M, Coccolini F, Labricciosa FM, Al-Hasan MN, Buonomo L, Cheadle WG, De Simone B, Duane TM, Hardcastle TC, Hecker A, Kirkpatrick AW, Leone M, Martin-Loeches I, Marwah S, Maves RC, Montravers P, Podda M, Rello J, Tattevin P, Tessier J, Tranà C, Ulrych J, Vallicelli C, Vigutto G, Watkins RR, Coimbra R, Catena F. Necrotizing soft-tissue infections survival guide in adult patients: A position statement by the Global Alliance for Infections in Surgery. J Trauma Acute Care Surg Epub 2025 Dec 17. 10.1097/TA.0000000000004833 10.1097/TA.0000000000004833 41417687

[34] Lauerman MH, Kolesnik O, Sethuraman K, Rabinowitz R, Joshi M, Clark E, Stein D, Scalea T, Henry S. Less is more? Antibiotic duration and outcomes in Fournier’s gangrene. J Trauma Acute Care Surg. 2017;83:443–8. 28538648 10.1097/TA.0000000000001562

[35] Suzuki H, Muramatsu K, Kubo T, Kawasaki M, Fujitani T, Tsukamoto M, Uchida S, Fujino Y, Matsuda S, Sakai A. Factors associated with mortality among patients with necrotizing soft tissue infections: An analysis of 4597 cases using the Diagnosis Procedure Combination Database. Int J Infect Dis. 2021;102:73–8. 33065296 10.1016/j.ijid.2020.10.019

[36] van Stigt S, Knubben M, Schrooten T, Tan E. Prognostic factors for mortality in 123 severe cases of necrotizing fasciitis in 5 hospitals in the Netherlands between 2003 and 2017. Eur J Trauma Emerg Surg. 2022;48:1189–95. 34046689 10.1007/s00068-021-01706-z PMC9001207

[37] Bonnel AR, Bunchorntavakul C, Reddy KR. Immune dysfunction and infections in patients with cirrhosis. Clin Gastroenterol Hepatol. 2011;9:727–38. 21397731 10.1016/j.cgh.2011.02.031

[38] Albillos A, Lario M, Álvarez-Mon M. Cirrhosis-associated immune dysfunction: distinctive features and clinical relevance. J Hepatol. 2014;61:1385–96. 25135860 10.1016/j.jhep.2014.08.010

[39] Bartoletti M, Giannella M, Lewis R, Caraceni P, Tedeschi S, Paul M, Schramm C, Bruns T, Merli M, Cobos-Trigueros N, Seminari E, Retamar P, Muñoz P, Tumbarello M, Burra P, Torrani Cerenzia M, Barsic B, Calbo E, Maraolo AE, Petrosillo N, Galan-Ladero MA, D’Offizi G, Bar Sinai N, Rodríguez-Baño J, Verucchi G, Bernardi M, Viale P. ESGBIS/BICHROME Study Group. A prospective multicentre study of the epidemiology and outcomes of bloodstream infection in cirrhotic patients. Clin Microbiol Infect. 2018;24:546.e1-546.e8. 10.1016/j.cmi.2017.08.001 10.1016/j.cmi.2017.08.001 28818628

[40] van Stigt SFL, Schrooten TKJ, Knubben M, Tan ECTH. Impact of severe necrotizing fasciitis on quality of life in the Netherlands. Eur J Trauma Emerg Surg. 2022;48:4805–11. 35678866 10.1007/s00068-022-02011-z PMC9712305

[41] Urbina T, Canoui-Poitrine F, Hua C, Layese R, Alves A, Ouedraogo R, Bosc R, Sbidian E, Chosidow O, Dessap AM, de Prost N, Henri Mondor Hospital Necrotizing Fasciitis Group. Long-term quality of life in necrotizing soft-tissue infection survivors: a monocentric prospective cohort study. Ann Intensive Care. 2021;11:102. 10.1186/s13613-021-00891-9. 34213694 10.1186/s13613-021-00891-9 PMC8253876

[42] Nawijn F, Kerckhoffs MC, Hietbrink F. Quality of Life After Intensive Care Unit Admittance for Necrotizing Soft Tissue Infections Is Deemed Acceptable for Patients. Surg Infect (Larchmt). 2023;24:924–9. 38032595 10.1089/sur.2023.184

