# Supplementary material for: Factors associated with in-hospital mortality in necrotising soft tissue infections. a multicentre retrospective cohort study
Source: Eur J Trauma Emerg Surg. 2026 Jun 12;52(1):187. doi: 10.1007/s00068-026-03242-0 (PMC13263272; doi:10.1007/s00068-026-03242-0)
Supplement: Supplementary file 1 — Supplementary Material 1 [file 68_2026_3242_MOESM1_ESM.docx]

**Supplementary Table 1.** Baseline characteristics of the study subpopulation of patients with necrotising fasciitis of the limbs.

| **Diagnosis** | **N.** | **Percentage** | **Missing data N. (%)** |
| --- | --- | --- | --- |
| Necrotising fasciitis of the limbs | N. 224 | % 59.1 |  |
|  |  |  |  |
| **Type of NSTI** |  |  |  |
| Type I | N. 120 | % 53.6 | - |
| Type II | N. 78 | % 34.8 | - |
| Type III | N. 10 | % 4.5 | - |
| Type IV | N. 16 | % 7.1 | - |
|  |  |  |  |
| **Microbial species** |  |  |  |
| Escherichia coli | N. 36 | % 16.1 | - |
| **Staphylococcus aureus** | N. 12 | % 5.4 | - |
| **Streptococcus anginosus group** | N. 28 | % 12.5 | - |
| **Enterococcus spp.** | N. 29 | % 12.9 | - |
| Klebsiella pneumoniae | N. 11 | % 4.9 | - |
| Pseudomonas aeruginosa | N. 17 | % 7.6 | - |
| Multiresistant spp. (MRSA, ESBL, KPC) | N. 14 | % 6.3 | - |
| Candida albicans | N. 12 | % 5.4 | - |
| Candida glabrata | N. 3 | % 1.3 | - |
|  |  |  |  |
| **Antibiotics used (in combination)** |  |  |  |
| Piperacillin-Tazobactam | N. 134 | % 59.8 | - |
| Linezolid | N. 47 | % 21.0 | - |
| Meropenem | N. 66 | % 29.5 | - |
| Clindamycin | N. 61 | % 27.2 | - |
| Daptomycin | N. 69 | % 30.8 | - |
| Metronidazole | N. 34 | % 15.2 | - |
| Vancomycin | N. 33 | % 14.7 | - |
| Amoxicillin/Clavulanic acid | N. 38 | % 17.0 | - |
| Ceftriaxone | N. 12 | % 5.4 | - |
| Teicoplanin | N. 9 | % 4.0 | - |
| Levofloxacin | N. 21 | % 9.4 | - |
| Colistin | N. 7 | % 3.1 | - |
| Ceftazidime/Avibactam | N. 4 | % 1.8 | - |
| Fluconazole | N. 14 | % 6.3 | - |
| Caspofungin | N. 7 | % 3.1 | - |
| Echinocandins | N. 1 | % 0.4 | - |
|  |  |  |  |
| **Age (years)** | Median 59.50 | 48.0-68.0 (IQR 20.2) | - |
| **Male sex** | N. 128 | % 57.1 | - |
| **Female sex** | N. 96 | % 47.9 | - |
| **Body Mass Index (BMI) Kg/m^2^** | Median 24.76 | 22.2-28.7 (IQR 6.5) | 3 (1.3%) |
|  |  |  |  |
| **Tobacco smoking (active)** | N. 62 | % 27.7 | - |
| **Alcohol consumption (active)** | N. 30 | % 13.3 | - |
| **Intravenous drug use (active)** | N. 25 | % 11.1 | - |
| **Arterial hypertensions** | N. 95 | % 42.4 | - |
| **Diabetes** | N. 76 | % 33.9 | - |
| **Ischaemic heart disease** | N. 39 | % 17.4 | - |
| **Peripheral neuropathy** | N. 14 | % 6.3 | - |
| **Active cancer disease** | N. 18 | % 8.0 | - |
| **Cirrhosis** | N. 16 | % 7.1 | - |
| **Chronic kidney disease** | N. 31 | % 13.8 | - |
| **Chronic liver disease** | N. 27 | % 12.0 | - |
| **Chronic respiratory failure** | N. 11 | % 4.9 | - |
| **Chronic cardiac failure** | N. 12 | % 5.4 | - |
| **Chronic obstructive pulmonary disease** | N. 11 | % 4.9 | - |
| **Dermatological disease** | N. 14 | % 6.3 | - |
| **Haematological malignancies** | N. 17 | % 7.6 | - |
| **Chronic corticosteroid therapy** | N. 26 | % 11.6 | - |
| **Ongoing hypoglycaemic therapy** |  |  |  |
| Oral hypoglycaemic therapy | N. 40 | % 17.9 | - |
| Insulin | N. 8 | % 3.6 | - |
| **Ongoing long-term NSAID therapy** | N. 18 | % 8.0 | - |
| **Recent surgery** | N. 29 | % 12.9 | - |
|  |  |  |  |
| **Duration of symptoms before hospital admission (days)** | Median 5.00 | 2.0-10.0 (IQR 8.0) | 14 (6.2%) |
| **Body temperature at admission (°C)** | Median 37.30 | 36.6-38.0 (IQR 1.4) | 1 (0.4%) |
| **Heart rate at admission (bpm)** | Median 96.00 | 80.0-110.0 (IQR 30.0) | 1 (0.4%) |
| **Respiratory rate at admission (breaths/min)** | Median 14.00 | 13.0-18.0 (IQR 5.0) | 31 (13.8%) |
| **Systolic blood pressure at admission (mmHg)** | Median 120.0 | 102.0-130.0 (IQR 27.7) | 2 (0.8%) |
| **Diastolic blood pressure at admission (mmHg)** | Median 70.70 | 60.0-79.0 (IQR 18.7) | 2 (0.8%) |
|  |  |  |  |
| **White Blood Cell (WBC) count at admission (x10^9/L)** | Median 14.08 | 8.6-20.5 (IQR 12.09) | 2 (0.8%) |
| **Haemoglobin at admission (g/dL)** | Median 11.20 | 9.4-13.0 (IQR 3.5) | - |
| **Glycaemia at admission (mg/dL)** | Median 123.5 | 96.0-181.0 (IQR 84.7) | - |
| **C-reactive protein (CRP) at admission (mg/L)** | Median 201.0 | 104.0-291.0 (IQR 189.0) | 13 (5.8%) |
| **Procalcitonin (PCT) at admission (ng/mL)** | Median 2.60 | 0.7-15.1 (IQR 14.8) | 77 (34.4%) |
| **Serum sodium at admission (mEq/L)** | Median 137.0 | 132.0-140.0 (IQR 8.0) | - |
| **Serum creatinine at admission (mg/dL)** | Median 0.99 | 0.6-1.6 (IQR 1.00) | - |
|  |  |  |  |
| **LRINEC score** | Median 6.00 | 3.0-8.0 (IQR 5.0) | 4 (1.6%) |
|  |  |  |  |
