# Supplementary material for: Factors associated with in-hospital mortality in necrotising soft tissue infections. a multicentre retrospective cohort study
Source: Eur J Trauma Emerg Surg. 2026 Jun 12;52(1):187. doi: 10.1007/s00068-026-03242-0 (PMC13263272; doi:10.1007/s00068-026-03242-0)
Supplement: Supplementary file 2 — Supplementary Material 2 [file 68_2026_3242_MOESM2_ESM.docx]

**Supplementary Table 2.** Baseline characteristics of the study subpopulation of patients with Fournier’s gangrene.

| **Diagnosis** | **N.** | **Percentage** | **Missing data N. (%)** |
| --- | --- | --- | --- |
| Fournier’s gangrene | N. 85 | % 22.4 |  |
|  |  |  |  |
| **Type of NSTI** |  |  |  |
| Type I | N. 56 | % 65.9 | - |
| Type II | N. 26 | % 30.6 | - |
| Type III | N. 3 | % 3.6 | - |
| Type IV | N. - | % - | - |
|  |  |  |  |
| **Microbial species** |  |  |  |
| Escherichia coli | N. 36 | % 42.4 | - |
| **Staphylococcus aureus** | N. 4 | % 4.7 | - |
| **Streptococcus anginosus group** | N. 15 | % 17.6 | - |
| **Enterococcus spp.** | N. 10 | % 11.8 | - |
| Klebsiella pneumoniae | N. 8 | % 9.4 | - |
| Pseudomonas aeruginosa | N. 7 | % 8.2 | - |
| Multiresistant spp. (MRSA, ESBL, KPC) | N. 5 | % 5.9 | - |
| Candida albicans | N. 5 | % 5.9 | - |
| Candida glabrata | N. 5 | % 5.9 | - |
|  |  |  |  |
| **Antibiotics used (in combination)** |  |  |  |
| Piperacillin-Tazobactam | N. 54 | % 63.5 | - |
| Linezolid | N. 12 | % 14.1 | - |
| Meropenem | N. 29 | % 34.1 | - |
| Clindamycin | N. 19 | % 22.4 | - |
| Daptomycin | N. 21 | % 24.7 | - |
| Metronidazole | N. 23 | % 27.1 | - |
| Vancomycin | N. 10 | % 11.8 | - |
| Amoxicillin/Clavulanic acid | N. 9 | % 10.6 | - |
| Ceftriaxone | N. 7 | % 8.2 | - |
| Teicoplanin | N. 3 | % 3.5 | - |
| Levofloxacin | N. 4 | % 4.7 | - |
| Colistin | N. - | % - | - |
| Ceftazidime/Avibactam | N. - | % - | - |
| Fluconazole | N. 6 | % 7.1 | - |
| Caspofungin | N. 3 | % 3.5 | - |
| Echinocandins | N. - | % - | - |
|  |  |  |  |
| **Age (years)** | Median 62.00 | 47.0-72.0 (IQR 25.0) | - |
| **Male sex** | N. 66 | % 77.6 | - |
| **Female sex** | N. 19 | % 22.4 | - |
| **Body Mass Index (BMI) Kg/m^2^** | Median 25.96 | 23.0-29.4 (IQR 6.35) | 2 (2.3%) |
|  |  |  |  |
| **Tobacco smoking (active)** | N. 27 | % 31.8 | - |
| **Alcohol consumption (active)** | N. 9 | % 10.6 | - |
| **Intravenous drug use (active)** | N. 5 | % 5.9 | - |
| **Arterial hypertensions** | N. 43 | % 50.6 | - |
| **Diabetes** | N. 41 | % 48.2 | - |
| **Ischaemic heart disease** | N. 18 | % 21.2 | - |
| **Peripheral neuropathy** | N. 5 | % 5.9 | - |
| **Active cancer disease** | N. 11 | % 12.9 | - |
| **Cirrhosis** | N. 8 | % 9.4 | - |
| **Chronic kidney disease** | N. 10 | % 11.8 | - |
| **Chronic liver disease** | N. 10 | % 11.8 | - |
| **Chronic respiratory failure** | N. 8 | % 9.4 | - |
| **Chronic cardiac failure** | N. 12 | % 14.1 | - |
| **Chronic obstructive pulmonary disease** | N. 1 | % 1.2 | 1 (1.2%) |
| **Dermatological disease** | N. 6 | % 7.1 | - |
| **Haematological malignancies** | N. 6 | % 7.1 | - |
| **Chronic corticosteroid therapy** | N. 4 | % 4.7 | - |
| **Ongoing hypoglycaemic therapy** |  |  |  |
| Oral hypoglycaemic therapy | N. 14 | % 16.5 | - |
| Insulin | N. 10 | % 11.8 | - |
| **Ongoing long-term NSAID therapy** | N. 9 | % 10.6 | - |
| **Recent surgery** | N. 11 | % 12.9 | - |
|  |  |  |  |
| **Duration of symptoms before hospital admission (days)** | Median 5.00 | 3.0-12.0 (IQR 9.0) | 7 (8.2%) |
| **Body temperature at admission (°C)** | Median 37.40 | 36.6-38.3 (IQR 1.75) | 1 (1.2%) |
| **Heart rate at admission (bpm)** | Median 92.00 | 83.0-110.0 (IQR 27.0) | - |
| **Respiratory rate at admission (breaths/min)** | Median 16.00 | 14.0-18.0 (IQR 4.0) | 3 (3.5%) |
| **Systolic blood pressure at admission (mmHg)** | Median 111.5 | 100.0-130.0 (IQR 30.0) | 2 (2.3%) |
| **Diastolic blood pressure at admission (mmHg)** | Median 66.00 | 60.0-75.0 (IQR 15.0) | 2 (2.3%) |
|  |  |  |  |
| **White Blood Cell (WBC) count at admission (x10^9/L)** | Median 15.00 | 10.3-20.3 (IQR 9.9) | 2 (2.3%) |
| **Haemoglobin at admission (g/dL)** | Median 11.40 | 9.6-13.2 (IQR 3.60) | - |
| **Glycaemia at admission (mg/dL)** | Median 133.00 | 9.8-13.4 (IQR 121.0) | - |
| **C-reactive protein (CRP) at admission (mg/L)** | Median 212.00 | 123.0-332.0 (IQR 209.0) | 9 (10.6%) |
| **Procalcitonin (PCT) at admission (ng/mL)** | Median 2.68 | 0.9-5.8 (IQR 4.8) | 8 (9.4%) |
| **Serum sodium at admission (mEq/L)** | Median 136.0 | 133.0-139.0 (IQR 6.0) | - |
| **Serum creatinine at admission (mg/dL)** | Median 1.10 | 0.9-2.0 (IQR 1.1) | - |
|  |  |  |  |
| **LRINEC score** | Median 6.50 | 4.0-8.0 (IQR 4.0) | 9 (10.6%) |
|  |  |  |  |
