# Supplementary material for: Factors associated with in-hospital mortality in necrotising soft tissue infections. a multicentre retrospective cohort study
Source: Eur J Trauma Emerg Surg. 2026 Jun 12;52(1):187. doi: 10.1007/s00068-026-03242-0 (PMC13263272; doi:10.1007/s00068-026-03242-0)
Supplement: Supplementary file 3 — Supplementary Material 3 [file 68_2026_3242_MOESM3_ESM.docx]

**Supplementary Table 3.** Predictors of in-hospital mortality in subpopulations.

|  | **Odds Ratio/Mean Difference** | **95%CI** | **P value** | **Multivariable analysis** |
| --- | --- | --- | --- | --- |
| ***Patients with necrotising fasciitis of the limbs*** |  |  |  |  |
| Age | 1.081 | 1.050–1.116 | <0.001 | aOR 1.079 per year, 95%CI 1.051–1.114, P< 0.001; Youden’s ≥ 61 years |
| Arterial hypertension | 2.777 | 1.348-5.718 | 0.006 |  |
| Diabetes | 2.263 | 1.114-4.595 | 0.024 |  |
| Ischaemic heart disease | 2.229 | 1.025-5.158 | 0.043 |  |
| Chronic kidney disease | 3.381 | 1.459-7.838 | 0.002 | aOR 2.885, 95%CI 1.141–7.283; P= 0.025 |
| Chronic cardiac failure | 5.625 | 1.707-18.535 | 0.005 |  |
| Haematological malignancies | 5.244 | 1.876-14.661 | 0.002 |  |
| **Respiratory rate at admission (breaths/min)** | 1.143 | 1.018–1.284 | 0.024 |  |
| Glycaemia at admission (mg/dL) | 1.003 | 1.000-1.005 | 0.046 | aOR 1.003, 95%CI 1.000–1.006; P= 0.080 |
| Serum creatinine at admission (mg/dL) | 1.389 | 1.112–1.734 | 0.004 | aOR 1.290, 95%CI 0.971–1.713; P= 0.079 |
|  |  |  |  |  |
| ***Patients with Fournier’s gangrene*** |  |  |  |  |
| Age | 1.074 | 1.030–1.124 | 0.002 | aOR 1.074 per year, 95%CI 1.030–1.124, P= 0.002; Youden’s ≥ 74 years |
| Tobacco smoking (active) | 0.193 | 0.041–0.907 | 0.037 |  |
| Chronic kidney disease | 7.154 | 1.765–28.990 | 0.006 | aOR 3.613, 95%CI 0.748–7.423, P= 0.110 |
|  |  |  |  |  |
